# Superficial white matter integrity in neuromyelitis optica spectrum disorder and multiple sclerosis

**DOI:** 10.1177/20552173231226107

**Published:** 2024-01-23

**Authors:** Darko Komnenić, Owen Robert Phillips, Shantanu H Joshi, Claudia Chien, Tanja Schmitz-Hübsch, Susanna Asseyer, Friedemann Paul, Carsten Finke

**Affiliations:** Charité–Universitätsmedizin Berlin, Corporate Member of Freie Universität Berlin and 9373 Humboldt-Universität zu Berlin, Berlin, Germany; 9373 Humboldt-Universität zu Berlin, Berlin School of Mind and Brain, Berlin, Germany; Division of Child and Adolescent Psychiatry, Department of Psychiatry, 10624 Stanford University School of Medicine, Stanford, CA, USA; Department of Neurology, Department of Bioengineering, 8783 University of California Los Angeles, Los Angeles, CA, USA; Charité–Universitätsmedizin Berlin, Corporate Member of Freie Universität Berlin and 9373 Humboldt-Universität zu Berlin & Max Delbrück Center for Molecular Medicine in the Helmholtz Association, Experimental and Clinical Research Center, Berlin, Germany; Charité–Universitätsmedizin Berlin, Corporate Member of Freie Universität Berlin and 9373 Humboldt-Universität zu Berlin, NeuroCure Clinical Research Center, Berlin, Germany; Department of Psychiatry and Neurosciences, Charité–Universitätsmedizin Berlin, corporate member of Freie Universität Berlin and 9373 Humboldt-Universität zu Berlin, Berlin, Germany; Charité–Universitätsmedizin Berlin, Corporate Member of Freie Universität Berlin and 9373 Humboldt-Universität zu Berlin & Max Delbrück Center for Molecular Medicine in the Helmholtz Association, Experimental and Clinical Research Center, Berlin, Germany; Charité–Universitätsmedizin Berlin, Corporate Member of Freie Universität Berlin and 9373 Humboldt-Universität zu Berlin, NeuroCure Clinical Research Center, Berlin, Germany; Charité–Universitätsmedizin Berlin, Corporate Member of Freie Universität Berlin and 9373 Humboldt-Universität zu Berlin & Max Delbrück Center for Molecular Medicine in the Helmholtz Association, Experimental and Clinical Research Center, Berlin, Germany; Max Delbrück Center for Molecular Medicine in the Helmholtz Association, Berlin, Germany; Department of Neurology, Charité–Universitätsmedizin Berlin, corporate member of Freie Universität Berlin and 9373 Humboldt-Universität zu Berlin, Berlin, Germany; 9373 Humboldt-Universität zu Berlin, Berlin School of Mind and Brain, Berlin, Germany; Department of Neurology, Charité–Universitätsmedizin Berlin, corporate member of Freie Universität Berlin and 9373 Humboldt-Universität zu Berlin, Berlin, Germany

**Keywords:** multiple sclerosis, neuromyelitis optica spectrum disorder, superficial white matter, mean diffusivity, diffusion tensor imaging, cognition, disability

## Abstract

**Background:**

Superficial white matter (SWM) is a particularly vulnerable area of white matter adjacent to cerebral cortex that was shown to be a sensitive marker of disease severity in several neurological and psychiatric disorders, including multiple sclerosis (MS), but has not been studied in neuromyelitis optica spectrum disorder (NMOSD).

**Objective:**

To compare the integrity of SWM between MS patients, NMOSD patients and healthy controls, and explore the correlation of SWM integrity with cognitive performance and overall disability.

**Methods:**

Forty NMOSD patients, 48 MS patients and 52 healthy controls were included in the study. Mean diffusivity (MD) values obtained by diffusion tensor imaging were used as a measure of SWM integrity. Cognitive performance and overall disability were assessed with standardized tests.

**Results:**

Superficial white matter MD was increased in MS patients compared to healthy controls. Higher MD was associated with poorer spatial memory (most prominently in right temporal and right limbic lobe) and poorer information processing speed in MS patients. After adjusting for age, no significant differences of SWM MD were observed between NMOSD patients and healthy controls.

**Conclusion:**

Integrity of SWM is compromised in MS, but not in NMOSD, and can serve as a sensitive marker of disease severity.

## Introduction

Neuromyelitis optica spectrum disorder (NMOSD) and multiple sclerosis (MS) are autoimmune demyelinating disorders of the central nervous system that can have a similar initial clinical presentation.^1^ However, while demyelination is a core hallmark of MS, in NMOSD it is a downstream, secondary phenomenon. In MS, white matter brain lesions exhibit specific lesion characteristics with regard to shape and distribution and have been incorporated into the diagnostic criteria.^2^ In NMOSD, brain magnetic resonance imaging (MRI) abnormalities are less prominent but have been reported in 50%–85% of patients.^3,4^

The distinction between NMOSD and MS is further supported by the results of studies using diffusion tensor imaging (DTI) to examine the integrity of deep white matter axonal pathways. Studies using DTI in MS reported white matter damage both within^5^ and beyond optic radiation.^6^ In NMOSD, the findings have been more varied with some studies only detecting significant damage in the optic radiation^7^ or associated regions,^8^ while others^9,10^ reported more diffuse and widespread damage in deep white matter. Studies of cortical myelin content showed extensive myelin loss in MS, especially in the progressive phases of the disease,^11^ and no cortical demyelination in aquaporin-4-antibody positive NMOSD.^12^ Using ultra-high field MRI, Sinnecker et al.^13^ also detected no pathological changes in cortices of NMOSD patients.

Superficial white matter (SWM) is the white matter at the interface of cortical gray matter and deep white matter.^14^ It mostly consists of short-range association fibers (U-fibers), and it is one of the last regions to myelinate.^15^ The oligodendrocytes in SWM, which develop later in life, are structurally more complex and metabolically overextended.^16^ Oligodendrocytes in deep white matter (types III and IV) usually wrap only one axon with about 100 layers, whereas in the SWM, oligodendrocytes (types I and II) may wrap up to 50 axonal segments with fewer than 10 myelin membrane layers.^16,17^ This makes the SWM an especially vulnerable area, because damage to only a small number of oligodendrocytes will have a much more detrimental effect than in deep white matter. However, these characteristics also suggest that the SWM is a promising region of interest for detecting early and subtle structural alterations. Indeed, recent studies have identified SWM damage in disorders believed to have no, or only mild structural brain damage, such as NMDAR encephalitis,^14^ schizophrenia, and bipolar disorder.^18^ This is further supported by a study by Buyukturkoglu et al.^19^ which reported SWM damage in early MS patients in areas underneath insula, inferior frontal, orbitofrontal, superior and medial temporal, and pre- and post-central cortices. However, the integrity of SWM has not been examined in NMOSD and compared to MS so far.

Here, we studied mean diffusivity (MD) as a measure of SWM integrity in MS patients, NMOSD patients, and healthy control participants (HCs). Given the prominent demyelination in MS, we expect more extensive SWM damage in MS patients, compared to NMOSD patients and HCs. In addition, we expect higher MD in NMOSD patients compared to HCs. In an exploratory analysis, we also examine the correlation of MD and the cognitive performance of NMOSD and MS patients, since higher MD has been shown to be associated with worse cognitive performance in other clinical entities.^14^

## Methods

### Study participants

Forty NMOSD patients fulfilling the 2015 International Diagnostic Criteria,^3^ 48 relapsing-remitting MS patients, fulfilling the 2017 revisions of the McDonald criteria,^2^ and 52 HCs were included in the study. Patients were recruited from the outpatient clinics of the NeuroCure Clinical Research Center and the Department of Neurology of Charité - Universitätsmedizin Berlin. Healthy control participants were volunteers with no history of neurological or psychiatric disorders, and matched in age to both patient groups and in sex to MS patients (Table 1). All NMOSD patients were seropositive for aquaporin-4-immunoglobulin-G antibodies (AQP4+) in a cell-based assay.^20^ The difference in sex composition between the three groups reflects the typical preponderance of female patients in AQP4+ cohorts.^21^ The study was approved by the ethics committee of Charité - Universitätsmedizin Berlin (EA1/163/12; EA1/077/11; EA1/189/13), and all participants gave written informed consent for data acquisition and publication.

**Table 1.** Demographic data of MS patients, NMOSD patients, and healthy controls (HC).

	MS	NMOSD	HC	Group difference
Age, range (years)	26–67	19–75	30–68	▪
Age, mean (SD)	45.9 (9.7)	48.2 (14.5)	44.1 (12.3)	*F*(2)= 1.27, *p *= 0.285
Sex ratio (F:M)	28:20	37:3	34:18	χ^2^(2)= 13.4, *p *=* *0*.*001
EDSS, mean (SD)	2.45 (1.53)	3.54 (1.76)	▪	t(79) = −2.99, *p *=* *0.004
Visual	0.53 (0.80)	1.79 (1.75)	▪	t(43) = −3.91, *p *=* *0.000
Brain Stem	0.72 (0.93)	0.50 (0.79)	▪	t(79) = 1.14, *p *=* *0.258
Pyramidal	1.19 (0.97)	1.15 (1.28)	▪	t(59) = 0.17, *p *=* *0.866
Cerebellar	0.79 (0.91)	0.21 (0.54)	▪	t(76) = 3.60, *p *=* *0.001
Sensory	0.96 (0.96)	0.50 (0.86)	▪	t(79) = 2.22, *p *=* *0.030
Bowel and Bladder	0.77 (0.89)	0.06 (0.34)	▪	t(63) = 4.96, *p *=* *0.000
Cerebral	0.68 (0.63)	0.12 (0.33)	▪	t(73) = 5.24, *p *=* *0.000
Disease duration in months, mean (SD)	158.42 (93.62)	101.71 (89.31)	▪	t(77) = −2.68, *p *= 0.009
Lesion volume in ml, mean (SD)	8.67 (8.99)	2.54 (3.44)	▪	t(63) = 4.35, *p *= 0.000
	Age difference between group pairs		Sex difference between group pairs	
MS vs NMOSD	t(86)= −0.89, *p *= .374		χ^2^(1) = 13.2, *p *<* *0*.*001	
HC vs NMOSD	t(90)= −1.46, *p *= .148		χ^2^(1) = 9.4, *p *=* *0*.*002	
HC vs MS	t(98)= −0.79, *p *= .431		χ^2^(1) = 0.53, *p *=* *0.468	

Both patient groups underwent neuropsychological testing on the day of MRI data acquisition, using the German translation of Brief Repeatable Battery of Neuropsychological Tests.^22^ The battery tests verbal (Selective Reminding Test) and spatial memory (10–36 Spatial Recall Test), information processing speed (Symbol Digit Modalities Test (SDMT)), sustained attention (Paced Auditory Serial Addition Test), and verbal fluency (Word List Generation (category: fruit and vegetables). The standardized scores on each subtest were calculated using the normative data provided in Scherer et al.,^22^ which take into account the patients’ age, education, and in some cases sex. In addition, the overall level of disability of patients was assessed using Expanded Disability Status Scale (EDSS).^23^ Table 1 shows the total EDSS scores, as well as scores on the EDSS’ functional subsystems for the two patient groups.

### Magnetic resonance imaging data acquisition and analysis

Magnetic resonance imaging data were acquired on a SIEMENS Tim Trio 3 T scanner, at Berlin Center for Advanced Neuroimaging, using the following sequences: (i) 3D magnetization-prepared rapid gradient echo sequence (1 × 1 × 1 mm resolution) and (ii) single-shot echo-planar imaging sequence for diffusion MRI acquisition (2.5 × 2.5 × 2.3 mm resolution). Please see the Appendix for additional parameters of the two sequences.

Magnetic resonance imaging data analysis was done as previously described.^14,15,24^ Briefly, T1-weighted images were processed with BrainSuite's (v 21a) cortical surface extraction pipeline to obtain surface models of the inner and outer boundaries of cerebral cortex for each participant. These surfaces were then registered to a reference atlas surface using BrainSuite's surface–volume registration software. The outputs of the surface-volume registration were visually inspected to ensure sufficient quality. The diffusion-weighted images were processed with BrainSuite's diffusion pipeline, which generated white matter meshes. The meshes for all subjects were aligned using BrainSuite's atlas reference, and the MD values were obtained using a 10-mm sphere which averaged the values around each mesh vertex.

Mean diffusivity values for the whole brain SWM were computed for each participant, initially on the entire SWM tissue. We then coregistered the map of white matter lesions for each participant to the white matter mesh image generated by BrainSuite. We used FSL's fslmaths function to exclude the areas of lesions from the image and computed the MD values for the resulting normal-appearing SWM once again. Since there were almost no lesions in the thin layer of SWM, the MD values obtained in the two analyses were very similar (Supplemental Figure 1). To check for between-group differences in global SWM MD, we performed *t*-tests in SPSS v20, as well as General Linear Model (GLM) analyses, with age and sex as covariates. In order to visualize the areas of the brain where the differences are most pronounced, we used BrainSuite to run a GLM at the vertex level.

Finally, we performed an exploratory set of correlation analyses between global measures of SWM and cognitive performance measures in patients. We also computed the correlations between MD and overall level of disability, represented by the score on EDSS. In addition, we calculated partial correlations, adjusting for age and sex. As with between-group comparisons, we visualized the significant correlations at the vertex level, using BrainSuite.

## Results

**Figure 1.**
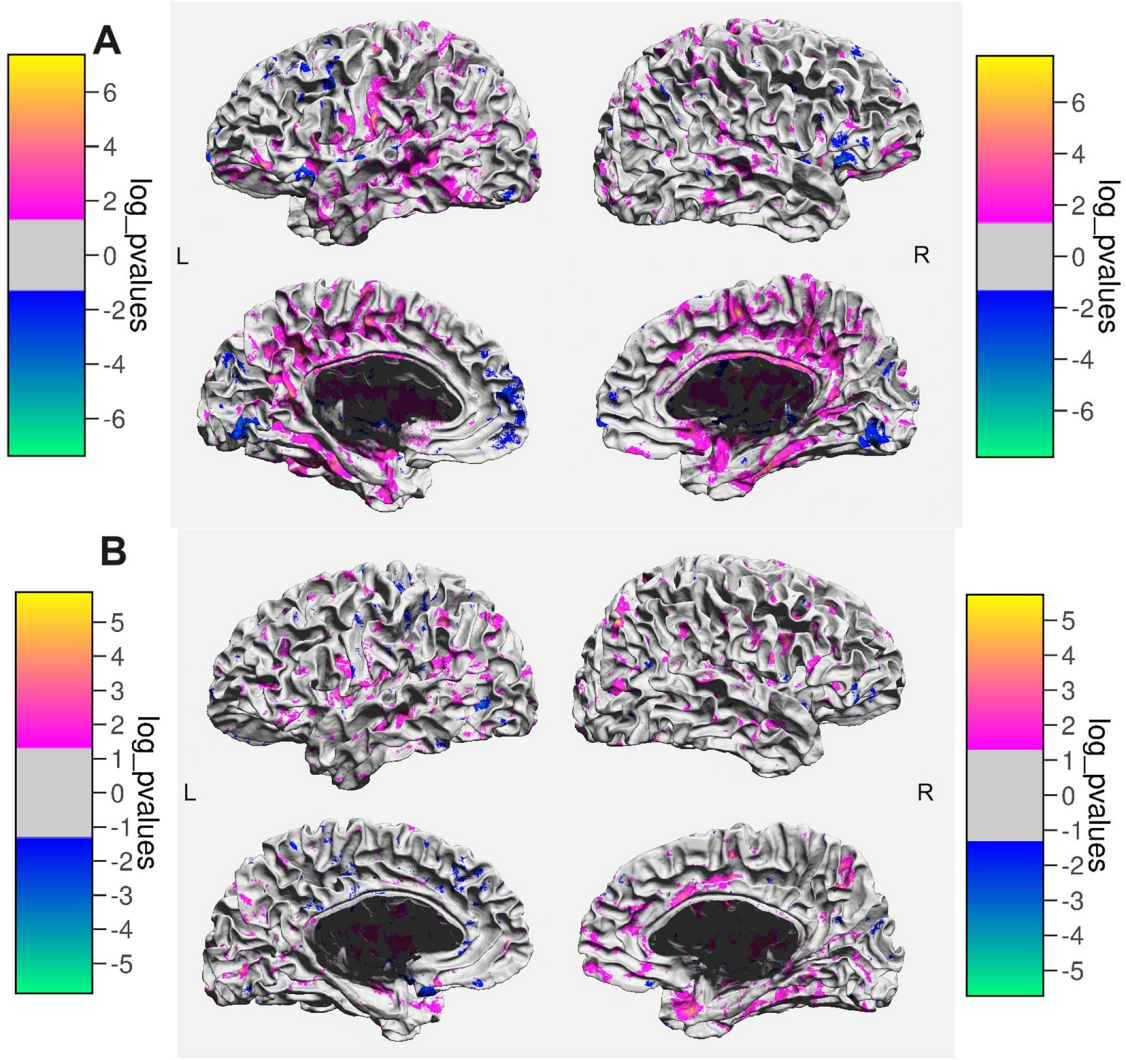
A) Areas showing differences in MD between MS patients and healthy controls. The color bars indicate the direction and the magnitude of the difference. B) Areas showing differences in MD between NMOSD patients and healthy controls. The color bars indicate the direction and the magnitude of the difference.

A *t*-test applied to whole brain SWM MD revealed an increase both in MS patients (t_HC−MS_(98) = −2.802, *p* = 0.006) and NMOSD patients (t_HC−NMOSD_(90) = −2.586, *p* = 0.011) in comparison to HCs, indicating impaired tissue integrity. There were no significant differences in the MD levels between MS and NMOSD patients (t_MS−NMOSD_(86) = 0.055, *p* = 0.957). After controlling for sex and age, the differences remained significant in the MS-HC comparison (*F*(1) = 7.664, *p* = 0.007), but not in the NMOSD-HC comparison (*F*(1) = 3.146, *p* = 0.080), where MD was more strongly predicted by age than by group status. Mapped across the brain surface, we observed widespread differences in comparisons of both patient groups with HCs (Figure 1A and B), with the difference being more prominent in the MS-HCs contrast.

**Figure 2.**
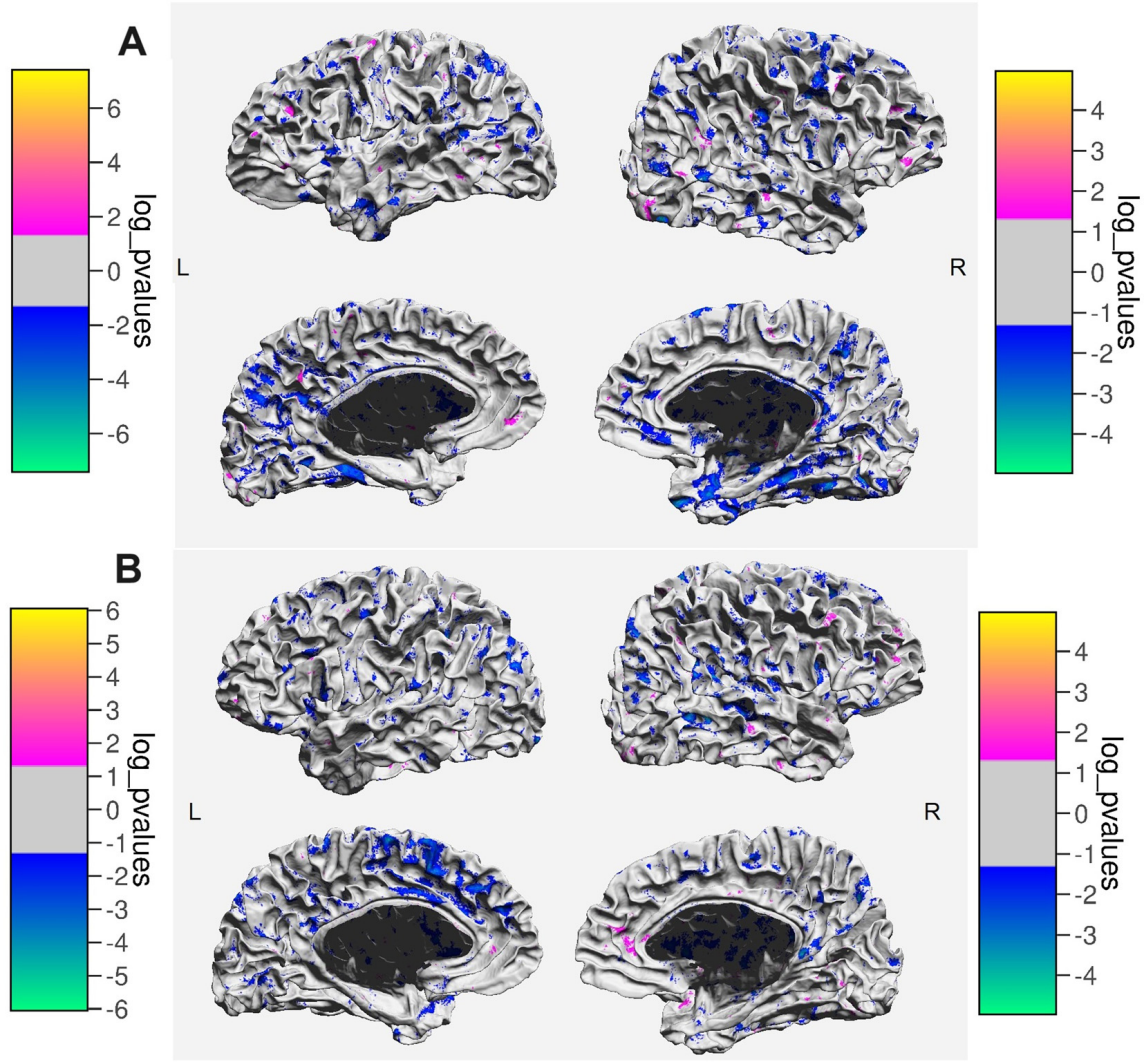
A) Areas showing correlations between MD and spatial delayed recall in MS patients. Higher scores on the test indicate better spatial memory. Color bars indicate the sign and the magnitude of the correlation. B) Areas showing correlations between MD and performance on Symbol Digit Modality Test in MS patients. Higher scores on the test indicate higher information processing speed and better sustained attention. Color bars indicate the sign and the magnitude of the correlation.

In MS patients, higher overall MD, indicating more prominent tissue alterations, was associated with poorer performance on spatial delayed recall and symbol-digit modality test (Table 2). These correlations remained significant after controlling for sex and age (Table 3). Mapping the correlations across the brain surface revealed that the negative association between MD and spatial delayed recall was most prominent in both limbic and temporal lobes (Figure 2A). For symbol-digit modality test, the association was strongest in the left medial frontal lobe (Figure 2B).

**Table 2.** Significant correlations obtained in the exploratory analysis of relationships between cognitive scores and MD in the two patient groups.

Group	Test	Area	Correlation
MS	zSPART-DR (spatial delayed recall)	Whole brain	*r* = −0.331, *p* = 0.028
		Right limbic lobe	*r* = −0.462, *p* = 0.002
		Right temporal lobe	*r* = −0.370, *p* = 0.013
	SDMT (information processing speed)	Whole brain	*r* = −0.315, *p* = 0.037
		Left parietal lobe	*r* = −0.376, *p* = 0.012
		Right parietal lobe	*r* = −0.368, *p* = 0.014
NMOSD	EDSS	Whole brain	*r* = 0.426, *p* = 0.012
	zSCLTR (verbal long-term memory)	Whole brain	*r* = −0.372, *p* = 0.030
		Left temporal lobe	*r* = −0.385, *p* = 0.025

**Table 3.** Partial correlations obtained in the exploratory analysis of relationships between cognitive scores and MD in the two patient groups, controlling for age and sex.

Group	Test	Area	Correlation
MS	zSPART-DR (spatial delayed recall)	Whole brain	*r* = −0.308, *p* = 0.047
		Right limbic lobe	*r* = −0.452, *p* = 0.003
		Right temporal lobe	*r* = −0.352, *p* = 0.022
	SDMT (information processing speed)	Whole brain	*r* = −0.312, *p* = 0.045
		Left parietal lobe	*r* = −0.376, *p* = 0.014
		Right parietal lobe	*r* = −0.370, *p* = 0.016
NMOSD	EDSS	Whole brain	*r* = 0.312, *p* = 0.082
	zSCLTR (verbal long-term memory)	Whole brain	*r* = −0.304, *p* = 0.091
		Left temporal lobe	*r* = −0.249, *p* = 0.170

In NMOSD patients, higher overall MD was associated with higher overall disability (EDSS) and poorer verbal memory performance (Table 2), but these correlations were no longer significant after controlling for sex and age (Table 3). Specifically, it was age that correlated significantly with MD in the NMOSD subsample (*r*(38) = .494, *p* = 0.001). A similar correlation was observed among the HCs (*r*(50) = .468, *p* = 0.000), but not among the MS patients (*r*(46) = .158, *p* = 0.283).

## Discussion

This study examined the integrity of the particularly vulnerable SWM in patients with MS and NMOSD. We observed increased MD, indicating SWM damage, in MS patients in comparison to HCs. Importantly, SWM MD correlated with measures of spatial memory and information processing speed in MS patients. These correlations remained significant after adjusting for age. In contrast, the differences in MD between NMOSD patients and HCs, as well as correlations of MD with cognitive scores and EDSS in NMOSD subsample, did not remain significant after adjusting for age. Age had a moderate and highly significant correlation with MD both in NMOSD patients and HCs, but not in MS patients. These results suggest that SWM MD changes in MS primarily reflect tissue damage. On the other hand, in NMOSD, a larger part of the variability of SWM MD is explained by normal aging.

Superficial white matter is the white matter situated directly below the cortical gray matter. Our findings suggest that SWM can be used as a sensitive measure for an early detection of brain damage in MS. In these patients, SWM MD holds the potential as an imaging marker to track disease trajectory and treatment response. In NMOSD, MD in the normal-appearing SWM was not significantly increased after correcting the results for age, which is in line with studies reporting preserved myelin content in the cortex of NMOSD patients.^12,13^ However, previous studies on deep white matter in NMOSD showed conflicting results with regard to impaired^9,25^ or preserved^7,8^ deep white matter. Given these conflicting results and our findings that show a trend for an increased SWM MD in NMOSD, it seems plausible that studies of larger patient samples or patients with more advanced disease stages will be able to detect significant SWM MD changes in NMOSD, albeit with a smaller effect size in comparison to MS.

The general potential of SWM analyses to detect subtle white matter alterations has been shown in several neuropsychiatric disorders. For example, Phillips et al.^14^ showed that fully recovered patients with anti-NMDA-receptor encephalitis had normal SWM diffusivity when compared to HCs, whereas patients with persisting cognitive impairment exhibited widespread SWM damage. In schizophrenia^26^ and bipolar disorder,^27^ the findings of damage in SWM complemented and expanded previous findings on damage in deep white matter and suggested that SWM lesions might be part of the structural basis for the signs and symptoms of the two diseases. In a study by Ji et al.,^18^ it was hypothesized that the overlapping patterns of SWM damage in schizophrenia and bipolar disorder could account for some of the shared features of the two diseases. These findings in other clinical entities can be used to direct future research efforts in NMOSD and MS, aimed at improving the understanding of the role of SWM in the development and manifestation of both disorders.

The finding that MS patients with more prominent SWM damage performed worse on the SDMT is consistent with the well-established view of the SDMT as a sensitive measure of cognitive impairment in MS.^28^ Moreover, higher damage in SWM correlated with poorer spatial delayed recall in MS. This is in line with previously reported cognitive deficits in MS^29^ and with the neuroanatomical organization of episodic memory.^30^ The negative correlation between spatial delayed recall and MD in MS patients is most prominent in right limbic and right temporal lobe, areas commonly associated with spatial memory.^31^

In MS, memory impairment has been attributed to damage of hippocampus, the key structure within medial temporal lobe, and to a degradation of its structural and functional connections.^32^ SWM, as the area directly below the cortex, contains both short-range and long-range axonal projections.^18^ Given the established role of the temporal lobe in memory formation and retrieval,^32^ damage to temporal lobe SWM might contribute to a disconnection between the hippocampus and other relevant nodes of the memory network.

Some potential limitations to our study are worth noting. Like with any method that segments brain tissue, partial volume effects can be a challenge. This is especially relevant in studies of the thin layer of SWM, given its complex structure that follows the folding of cerebral cortex. However, each image was visually inspected for quality control of the segmentation process and corrections were made when needed. Another limitation is the cross-sectional and correlational nature of our design which does not provide insight into causal relationships or the order in which changes occur. Further, the MRI was acquired without gadolinium contrast agent, and thus, information on disease activity was limited, and we could not compare MD between patients with active inflammation and those with older lesions. Future studies should also acquire functional connectivity sequences that will allow to better understand the functional consequences of SWM tissue damage.

In conclusion, our study observed an increase in MD of SWM in MS patients when compared to HCs, and no significant increase in NMOSD patients.

Our findings suggest that SWM MD could serve as a sensitive marker of tissue damage in MS. Future studies should replicate our results and the correlations of overall disability and cognitive scores with SWM damage in larger, independent samples.

## Supplemental Material

sj-docx-1-mso-10.1177_20552173231226107 - Supplemental material for Superficial white matter integrity in neuromyelitis optica spectrum disorder and multiple sclerosis Click here for additional data file. Supplemental material, sj-docx-1-mso-10.1177_20552173231226107 for Superficial white matter integrity in neuromyelitis optica spectrum disorder and multiple sclerosis by Darko Komnenić, Owen Robert Phillips, Shantanu H Joshi, Claudia Chien, Tanja Schmitz-Hübsch, Susanna Asseyer, Friedemann Paul and Carsten Finke in Multiple Sclerosis Journal – Experimental, Translational and Clinical

sj-jpg-2-mso-10.1177_20552173231226107 - Supplemental material for Superficial white matter integrity in neuromyelitis optica spectrum disorder and multiple sclerosis Click here for additional data file. Supplemental material, sj-jpg-2-mso-10.1177_20552173231226107 for Superficial white matter integrity in neuromyelitis optica spectrum disorder and multiple sclerosis by Darko Komnenić, Owen Robert Phillips, Shantanu H Joshi, Claudia Chien, Tanja Schmitz-Hübsch, Susanna Asseyer, Friedemann Paul and Carsten Finke in Multiple Sclerosis Journal – Experimental, Translational and Clinical
