# Supplementary material for: Superficial white matter integrity in neuromyelitis optica spectrum disorder and multiple sclerosis
Source: Mult Scler J Exp Transl Clin. 2024 Jan 23;10(1):20552173231226107. doi: 10.1177/20552173231226107 (PMC10807332; doi:10.1177/20552173231226107)
Supplement: sj-docx-1-mso-10.1177_20552173231226107 - Supplemental material for Superficial white matter integrity in neuromyelitis optica spectrum disorder and multiple sclerosis [file sj-docx-1-mso-10.1177_20552173231226107.docx]

MS Journal Appendix for MRI methodology

| **Hardware** | |
| --- | --- |
| Field strength | 3T |
| Manufacturer | SIEMENS |
| Model | TrioTim |
| Coil type  (e.g. head, surface) | Head |
| Number of coil channels | 12 |

| **Acquisition sequence** | | |
| --- | --- | --- |
| Type  (e.g. FLAIR, DIR, DTI, fMRI) | MPRAGE;  EPI | |
| Acquisition time | For MPRAGE: 04:24  For EPI: 08:32 | |
| Orientation | For MPRAGE: Sag>Cor(-11.3)>Tra(0.2)  For EPI: Tra>Cor(-3.4)>Sag(3.3) | |
| Alignment  (e.g. anterior commissure/poster commissure line) | AC-PC auto align | |
| Voxel size | For MPRAGE: 1*1*1mm  For EPI: 2.5*2.5 mm , Slice thickness: 2.3mm | |
| TR | For MPRAGE: 1900 ms  For EPI: 7500 ms | |
| TE | For MPRAGE: 3.03  For EPI: 86 | |
| TI | For MPRAGE: 900  For EPI: / | |
| Flip angle | For MPRAGE: 9  For EPI: 90 | |
| NEX | For MPRAGE: 2 | |
| Field of view | For MPRAGE: 256*256  For EPI: 192*192 | |
| Matrix size | For MPRAGE: 256*256  For EPI: 96*96 | |
| Parallel imaging | **Yes** | No |
| If used, parallel imaging method:  (e.g. SENSE, GRAPPA) | For MPRAGE: None  For EPI: GRAPPA (R = 2) | |
| Cardiac gating | Yes | **No** |
| If used, cardiac gating method:  (e.g. PPU or ECG) |  | |
| Contrast enhancement | Yes | **No** |

| **Acquisition sequence** | |
| --- | --- |
| If used, provide name of contrast agent, dose and timing of scan post-contrast administration | / |
| Other parameters: |  |

| **Image analysis methods and outputs** | |
| --- | --- |
| ***Lesions*** | |
| Type  (e.g. Gd-enhancing, T2-hyperintense, T1-hypointense) |  |
| Analysis method |  |
| Analysis software |  |
| Output measure  (e.g. count or volume [ml]) |  |
| ***Tissue volumes*** | |
| Type  (e.g. whole brain, grey matter, white matter, spinal cord) |  |
| Analysis method |  |
| Analysis software |  |
| Output measure  (e.g. absolute tissue volume in ml, tissue volume as a fraction of intracranial volume, percentage change in tissue volumes) |  |
| ***Tissue measures (e.g. MTR, DTI, T1-RT, T2-RT, T2*, T2’, ^1^H-MRS, perfusion, Na)*** | |
| Type  (e.g. whole brain, grey matter, white matter, spinal cord, normal-appearing grey matter or white matter) | Superficial white matter |
| Analysis method | DTI |
| Analysis software | BrainSuite Diffusion Pipeline |
| Output measure | Mean diffusivity estimation |
| ***Other MRI measures (e.g. functional MRI)*** | |
| Type  (e.g. whole brain, grey matter, white matter, spinal cord, normal-appearing grey matter or white matter) |  |
| Analysis method |  |
| Analysis software |  |
| Output measure |  |

**Other analysis details:**
