# Supplementary figures and images for: Superficial white matter integrity in neuromyelitis optica spectrum disorder and multiple sclerosis
Source: Mult Scler J Exp Transl Clin. 2024 Jan 23;10(1):20552173231226107. doi: 10.1177/20552173231226107 (PMC10807332; doi:10.1177/20552173231226107)

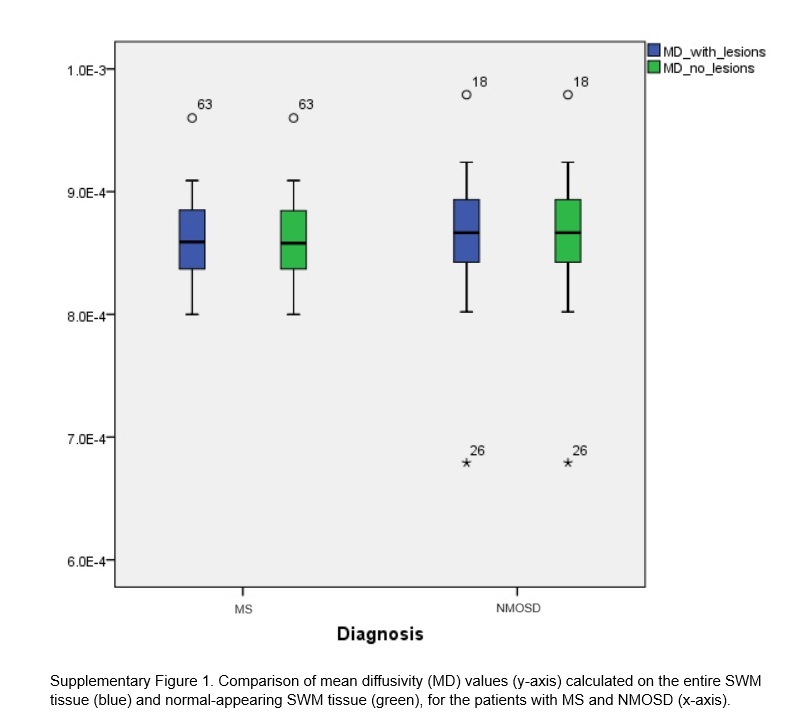

Supplement: sj-jpg-2-mso-10.1177_20552173231226107 - Supplemental material for Superficial white matter integrity in neuromyelitis optica spectrum disorder and multiple sclerosis [file sj-jpg-2-mso-10.1177_20552173231226107.jpg]
